# Fabrication and Characterization of an Electrochemical Platform for Formaldehyde Oxidation, Based on Glassy Carbon Modified with Multi-Walled Carbon Nanotubes and Electrochemically Generated Palladium Nanoparticles

**DOI:** 10.3390/ma17040841

**Published:** 2024-02-09

**Authors:** Andrzej Leniart, Barbara Burnat, Mariola Brycht, Maryia-Mazhena Dzemidovich, Sławomira Skrzypek

**Affiliations:** 1 University of Lodz, Faculty of Chemistry, Department of Inorganic and Analytical Chemistry, Tamka 12, 91-403 Lodz, Poland; barbara.burnat@chemia.uni.lodz.pl (B.B.); mariola.brycht@chemia.uni.lodz.pl (M.B.); maryia.mazhena.dzemidovich@edu.uni.lodz.pl (M.-M.D.); slawomira.skrzypek@chemia.uni.lodz.pl (S.S.); 2 University of Lodz, Doctoral School of Exact and Natural Sciences, Matejki 21/23, 90-231 Lodz, Poland

**Keywords:** electrocatalysis, atomic force microscopy, scanning electron microscopy, voltammetry, electrochemical impedance

## Abstract

This study outlines the fabrication process of an electrochemical platform utilizing glassy carbon electrode (GCE) modified with multi-walled carbon nanotubes (MWCNTs) and palladium nanoparticles (PdNPs). The MWCNTs were applied on the GCE surface using the drop-casting method and PdNPs were produced electrochemically by a potentiostatic method employing various programmed charges from an ammonium tetrachloropalladate(II) solution. The resulting GCEs modified with MWCNTs and PdNPs underwent comprehensive characterization for topographical and morphological attributes, utilizing atomic force microscopy and scanning electron microscopy along with energy-dispersive X-ray spectrometry. Electrochemical assessment of the GCE/MWCNTs/PdNPs involved cyclic voltammetry (CV) and electrochemical impedance spectroscopy conducted in perchloric acid solution. The findings revealed even dispersion of PdNPs, and depending on the electrodeposition parameters, PdNPs were produced within four size ranges, i.e., 10–30 nm, 20–40 nm, 50–60 nm, and 70–90 nm. Additionally, the electrocatalytic activity toward formaldehyde oxidation was assessed through CV. It was observed that an increase in the size of the PdNPs corresponded to enhanced catalytic activity in the formaldehyde oxidation reaction on the GCE/MWCNTs/PdNPs. Furthermore, satisfactory long-term stability over a period of 42 days was noticed for the GCE/MWCNTs/PDNPs(100) material which demonstrated the best electrocatalytic properties in the electrooxidation reaction of formaldehyde.

## 1. Introduction

Depleting reserves of fossil fuels, coupled with their negative environmental impact on one hand and increased demand for bioenergy and biofuels on the other, have prompted the exploration of new electrochemical platforms with extensive applications in fuel cells, catalysts, and electrochemical sensors. A specific emphasis is placed on the electrocatalytic oxidation of small organic compounds, including methanol, ethanol, isopropanol, formaldehyde, or formic acid, on various modified electrodes. This is due to their potential as electron donors in fuel cells, enabling the creation of high-power density systems [1]. Although direct applicability of formaldehyde in fuel cells is limited, its electrochemical oxidation plays a crucial role in understanding methanol oxidation, where formaldehyde serves as an intermediate [2]. Moreover, owing to the toxicity of formaldehyde and associated health risks, including chronic inflammation, vomiting, seizures, fetal development problems, cardiovascular diseases, and cancer [3,4,5,6,7], the detection and monitoring of its presence are of high importance.

Various electrochemical platforms, employing metals such as platinum, rhodium, nickel, and palladium as key components, have been utilized for the study of formaldehyde electrooxidation and detection [1,8,9,10,11,12,13,14,15,16,17,18,19,20]. Pd is particularly intriguing due to its distinctive physicochemical properties, especially its electrochemical attributes, ascribed to weak intermetallic bonds within the crystal lattice [21]. In addition, Pd demonstrates high stability and a remarkable tolerance to CO, which is formed as a byproduct during the oxidation of formaldehyde [22]. Unique catalytic properties of Pd contribute to its widespread use in both heterogeneous and homogeneous catalysis [23]. Pd serves as a catalyst in diverse processes, including the hydrogenation of alkene to alkanes and the oxidation of various organic compounds, with applications in both liquid and gaseous phases [24,25,26,27,28]. Additionally, it plays a crucial role in environmental technologies, acting as a catalyst for the combustion of petroleum products in automobile engines, facilitating hydrogenation reactions, and serving as a precursor in metallization processes for non-metallic materials such as fiberglass and ceramics used in electronics [29,30,31]. The versatility of Pd extends to its use in various types of sensors [32,33,34,35,36,37,38,39,40]. The production of Pd nanoparticles (PdNPs) employs various methods, encompassing physical techniques like physical vapor deposition and magnetron sputtering, chemical methods such as chemical vapor deposition and chemical reduction of metal ions, as well as electrochemical deposition [38,41,42].

It is important to emphasize that the choice of substrate onto which PdNPs are deposited significantly influences the effectiveness of its electrocatalytic activity. One such substrate is carbon, which plays a crucial role as the foundation for fuel cells, catalysts, and electrodes, highlighting its wide application in the field of energy and chemical processes. From an electrochemical perspective, the primary and preferred electrode material is glassy carbon (GC), renowned for its unique properties, including high electrical and thermal conductivity, hardness, resistance to extreme temperatures and chemicals, and stability at various polarization potentials [43,44,45,46]. To enhance existing properties or discover new ones for glassy carbon electrode (GCE), various modifications are applied using different techniques and materials. Carbon nanomaterials, known for their unique properties, are often employed for these modifications. They not only increase the surface area but also provide a substructure/substrate for subsequent materials used in the modification of electrochemical platforms. Carbon nanotubes (CNTs), specifically single- or multi-walled structures (SWCNTs or MWCNTs), are commonly used as carbon nanomaterials for surface modification [47,48,49,50] due to their unique features such as large specific surface area, high electrical and thermal conductivity, and substantial high mechanical strength [51]. Despite the superior properties of CNTs, they do not exhibit electrocatalytic activity toward formaldehyde oxidation; therefore, further modifications are required [52].

To date, the modification of CNTs with PdNPs to obtain a CNT/PdNP composite has been achieved through various methods, including chemical reduction of Pd(II) ions with sodium borohydride [13,53], formaldehyde [52,54], and sodium citrate in an ethylene glycol solution [55]. Other techniques involve the thermal decomposition of palladium acetate [39], RF magnetron sputtering [56], and electrochemical reduction of the MWCNT/lignosulfonate–Pd^2+^ composite [57]. However, these methods are time-consuming and involve multiple steps that are challenging to control, consequently affecting the final properties and the cost of the obtained electrode materials. As an alternative, one can propose an approach employing the electrochemical reduction of Pd(II) ions with charge control, offering full and precise control over the production of PdNPs, overcoming the challenges associated with time-consuming and complex procedures.

The objective of this work is to create an electrochemical platform of GCE/MWCNTs/PdNPs that exhibits improved electrocatalytic properties through the synergistic effect of MWCNTs and PdNPs. Significantly, the focus extends beyond the creation of the platform. This research places crucial importance on understanding and refining the straightforward electrochemical procedure of PdNP production, emphasizing controlled and precise nanoparticle synthesis. Additionally, the study aims to comprehensively characterize the morphology, topography, and elemental composition of the surfaces of the fabricated platforms, along with assessing their electrochemical and electrocatalytic properties in the electrooxidation reaction of formaldehyde.

## 2. Materials and Methods

### 2.1. Chemical Reagents

All reagents utilized in the experiments were of analytical purity and did not undergo additional purification processes. Solutions were prepared using triple-distilled water. The following solutions were employed for the measurements:

- hydrochloric acid solution (HCl, 35–38%, Avantor Performance, Gliwice, Poland) at a concentration of 0.1 mol L^−1^, containing ammonium tetrachloropalladate(II) ((NH_4_)_2_PdCl_4_, Ventron GMBH, Karlsruhe, Germany) at a concentration of 1.0 mmol L^−1^;
- perchloric acid solution (HClO_4_, 95%, Avantor Performance) at a concentration of 0.1 mol L^−1^ both without and with the addition of formaldehyde (HCHO, 37%, Avantor Performance) at a concentration of 0.1 mol L^−1^.

Prior to each measurement, the solutions underwent deoxygenation using argon with a rating of 5.0 (Linde Gaz Poland, Kraków, Poland).

### 2.2. Preparation of Electrodes

Six types of working electrodes were employed in the study, i.e., a glassy carbon electrode (GCE, diameter of 3 mm, L-Chem, Horka nad Moravou, Czechia), a GCE modified with multi-walled carbon nanotubes (GCE/MWCNTs), and four modified variations of GCE/MWCNTs containing different amounts of PdNPs (GCE/MWCNTs/PdNPs).

The preparation of the GCE surface involved manual polishing on felt using an aqueous Al_2_O_3_ suspension (ATM GMBH, Blieskastel, Germany) with a grain size of 0.3 µm. Following this, the electrode surface was rinsed with triple-distilled water, underwent a 5 min cleaning cycle in an ultrasonic cleaner, and was finally dried under an argon stream.

For the preparation of a GCE modified with MWCNTs (>95%, diameter 8–9 nm, length 5 µm, Sigma-Aldrich, Poznań, Poland) by the drop-drying method, a suspension of the MWCNTs in dimethylformamide (DMF, 99.8%, Avantor Performance, Gliwice, Poland) at a concentration of 0.5 mg L^−1^ was used. For this purpose, 5 µL of the MWCNT suspension was dropped onto the surface of the GCE and then the coated electrode was dried for 24 h, enabling the formation of a durable MWCNT layer. The GCE/MWCNTs underwent subsequent modification with PdNPs via electrochemical deposition from a 0.1 mol L^−1^ hydrochloric acid solution containing 1.0 mmol L^−1^ (NH_4_)_2_PdCl_4_. The PdNP synthesis was carried out using the coulometric method at a constant polarization potential, applying programmed and defined charge values of −5, −20, −50, and −100 mC. The deposition potential of 0.1 V ensured that the charge flowing during electrolysis was solely associated with the reduction reaction of Pd(II) ions to metallic Pd. Following the PdNP deposition process, each of the resulting GCE/MWCNTs/PdNPs underwent rinsing with triple-distilled water and drying using an argon stream. This procedure generated four variations of GCE/MWCNTs/PdNPs, each featuring different PdNPs contents, denoted as GCE/MWCNTs/PdNPs(5), GCE/MWCNTs/PdNPs(20), GCE/MWCNTs/PdNPs(50) and GCE/MWCNTs/PdNPs(100), containing 0.3, 1.1, 2.8, and 5.5 µg of metallic Pd, respectively (the mass of Pd was calculated based on Faraday’s laws).

### 2.3. The Research Methodology

The research strategy involved two primary approaches. The first approach focused on analyzing the topography and surface morphology of the working electrodes, along with elemental analysis of the fabricated GCE/MWCNTs/PdNPs. Characterization of the working electrodes involved atomic force microscopy (AFM) and scanning electron microscopy (SEM) coupled with energy dispersive X-ray spectrometry (EDX). AFM analyses were conducted utilizing a Dimension Icon system (Bruker, Santa Barbara, CA, USA), employing the intermittent contact method (tapping mode). A commercial TESPA V2 silicon probe (Bruker, Santa Barbara, CA, USA) with a nominal spring constant of 42 N m^−1^ and a resonance frequency of 320 kHz served as the AFM probe. SEM measurements were performed using a FEI Nova NanoSEM 450 microscope (FEI, Hillsboro, OR, USA), equipped with an electron gun with thermal field emission (Schottky emitter). SEM measurements were conducted with an accelerating voltage of 10 kV using a through-the-lens detector (TLD). Elemental surface composition was determined from EDX spectra collected with an EDX analyzer (Ametek Inc., Berwyn, PA, USA).

The second approach involved conducting an electrochemical evaluation of the working electrodes. This encompassed their characterization in a HClO_4_ solution using cyclic voltammetry (CV) and electrochemical impedance spectroscopy (EIS), as well as determining the electrocatalytic properties of GCE/MWCNT/PdNPs in the formaldehyde electrooxidation process. All electrochemical tests were carried out in a 15 mL electrochemical cell using a classic three-electrode configuration, where the working electrode was either GCE, GCE/MWCNTs, or GCE/MWCNTs/PdNPs with varying PdNPs contents, a silver chloride electrode (Ag|AgCl in 3 mol L^−1^ KCl solution, Mineral, Łomianki-Sadowa, Poland) served as the reference electrode, while a platinum wire (99.99%, The Mint of Poland, Warsaw, Poland) was used as an auxiliary electrode. All potential values in this work are referenced to Ag|AgCl. The electrochemical measurements were conducted using a PGSTAT 128 N potentiostat/galvanostat (Metrohm AUTOLAB B.V., Utrecht, The Netherlands), equipped with a frequency response module (FRA2) for electrochemical impedance spectroscopy measurements. Additionally, an M164 type electrode stand (MTM Anko Instruments, Kraków, Poland) was employed. The cyclic voltammograms were recorded in HClO_4_ solution, in both the presence and absence of formaldehyde, using a scan rate of 100 mV s^−1^. The EIS spectra were obtained over a frequency range of 10,000–0.01 Hz with an amplitude of 10 mV and 50 measuring points.

## 3. Results

### 3.1. Microscopic Characterization of the Working Electrodes

**Figure 1:**
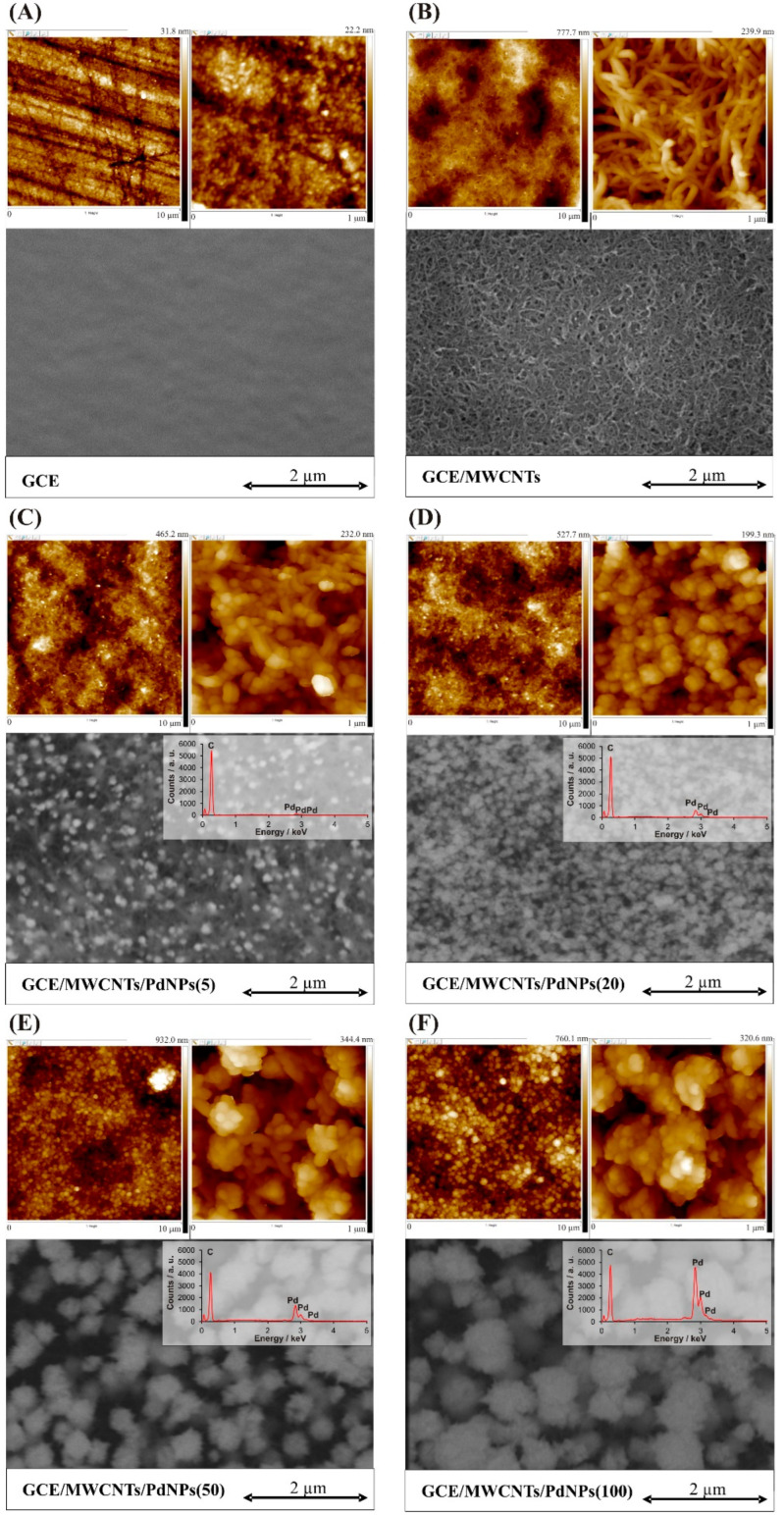
AFM (10 µm^2^ and 1 µm^2^ scanning areas) and SEM images (magnitude 100,000×) of the (**A**) GCE, (**B**) GCE/MWCNTs, (**C**) GCE/MWCNTs/PdNPs(5), (**D**) GCE/MWCNTs/PdNPs(20), (**E**) GCE/MWCNTs/PdNPs(50), and (**F**) GCE/MWCNTs/PdNPs(100) surfaces. Insets display EDX spectra for the electrodes with PdNPs.

**Figure 2:**
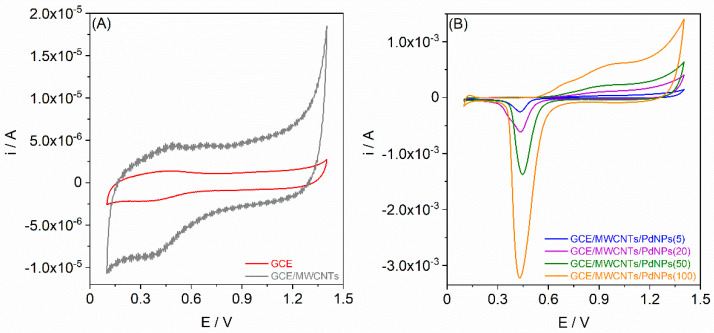
Cyclic voltammograms (scan rate of 100 mV s^−1^) recorded in 0.1 mol L^−1^ HClO_4_ solution for (**A**) the GCE and GCE/MWCNTs, as well as (**B**) the GCE/MWCNTs/PdNPs.

The assessment of topography and surface morphology for each type of working electrode, along with additional elemental analysis for GCE/MWCNTs/PdNPs with various PdNPs content, was conducted at three randomly selected places on the electrode surface. Figure 1 displays representative images depicting surface topography (AFM) and surface morphology (SEM) for the GCE and the GCE/MWCNTs, while Figure 2 presents AFM and SEM images of GCE/MWCNTs modified with different PdNPs contents (GCE/MWCNTs/PdNPs(5), GCE/MWCNTs/PdNPs(20), GCE/MWCNTs/PdNPs(50), and GCE/MWCNTs/PdNPs(100)).

The findings from both AFM and SEM images reveal distinct differences in surface topography and morphology among the examined electrodes. Notably, the unmodified GCE (Figure 1A) displays slight surface irregularities, probably stemming from polishing, evident as small scratches with depths measuring a few nanometers. At higher magnifications, the fine-grained structure of the GCE surface becomes evident. Moreover, the GCE surface appears homogeneous and clean.

In the case of GCE/MWCNTs (Figure 1B), the surface of the GCE appears to be entirely covered with multi-walled carbon nanotubes (MWCNTs) that are bent and intertwined. Additionally, areas containing a higher quantity of carbon nanotubes forming aggregates can be observed. The generated layer of MWCNTs on the surface of the glassy carbon electrode leads to a significant increase in surface area, as indicated by the height scale in the AFM images.

SEM and AFM images of the GCE/MWCNTs/PdNPs (Figure 1C–F) clearly illustrate both CNTs and electrodeposited PdNPs on the surface. In the case of GCE/MWCNTs/PdNPs(5) (Figure 1C), where the smallest amount of Pd (0.3 µg) was deposited, visible PdNPs measuring between 10 nm to 30 nm are observed. With a larger amount of deposited Pd (1.1 µg) on the GCE/MWCNTs/PdNPs(20), larger and more densely packed PdNPs are formed (Figure 1D), ranging from 20 nm to 40 nm. Following the deposition of 2.8 µg and 5.5 µg of Pd, subsequent AFM and SEM images of the GCE/MWCNTs/PdNPs(50) (Figure 1E) and GCE/MWCNTs/PdNPs(100) (Figure 1F) demonstrate the formation of even larger PdNPs. In the case of GCE/MWCNTs/PdNPs(50), the PdNPs measure between 50 nm and 60 nm, while for GCE/MWCNTs/PdNPs(100), their size ranges from 70 nm to 90 nm.

Analysis of the results indicates that the nucleation and further growth of PdNPs are prominently observed during the initial stages of electrodeposition, primarily occurring on CNTs until they are fully coated. Subsequent electrodeposition processes primarily contribute to the enlargement of these nanoparticles. The applied procedure of electrochemical generation of PdNPs leads to obtaining nanoparticles that should be considered as larger particles than clusters [58].

To analyze the surfaces of the tested electrodes using AFM images with scanning areas of 1 µm^2^, topographic parameters were determined utilizing the AFM image analysis program NanoScope Analysis v. 1.50 (Bruker). These parameters included real surface area (Sr; derived from AFM images), surface area difference (SAD; ratio of the geometric surface area (scanning area) to the surface area obtained from three-dimensional (3D) AFM images, expressed as a percentage), root mean square roughness coefficient (Rq; the mean square deviation between points on the tested surface and those on the optimal plane), average roughness coefficient (Ra; the arithmetic mean values of absolute deviations between points on the tested surface and those on the optimal plane), and the maximum height (Rmax; the vertical distance between the highest and lowest data points on the tested surface area after to the plane fit operation). The values of these parameters are detailed in Table 1.

Upon analyzing the determined topographic parameters outlined in Table 1, it is evident that the application of MWCNTs onto the GCE surface amplified all its topographic parameters. The roughness coefficients, Rq and Ra, underwent a 10-fold increase, while the SAD escalated by almost 40 times. The deposition of a small quantity of PdNPs, specifically 0.3 and 1.1 µg, reduced the topographic parameters compared with the GCE/MWCNTs. This occurred due to the partial filling of holes (gaps) between entangled nanotubes by the PdNPs, resulting in a partial smoothing of the surface. However, the deposition of larger amounts of Pd led to an increase in topographic values that is attributed to the formation of progressively larger PdNPs on the surface of the GCE/MWCNTs (Figure 1E,F). It can be stated that the deposition of Pd under the conditions applied by us leads to the formation PdNPs of various sizes, which can be easily controlled by adjusting the value of the charge during electrodeposition.

Additionally, elemental analysis was performed for the GCE/MWCNTs/PdNPs to confirm that the presence of PdNPs and to determine the Pd content using a standard-free quantitative analysis. The outcomes of the quantitative elemental composition for each of the GCE/MWCNTs/PdNPs are detailed in Table 2.

### 3.2. Electrochemical Characterization of the Working Electrodes

Cyclic voltammograms for each electrode were recorded in a 0.1 mol L^−1^ HClO_4_ solution, spanning a potential range from +0.1 V to +1.4 V, using a potential scan rate of 100 mV s^−1^ (Figure 2).

As can clearly be seen from Figure 2A, the CV voltammograms for both the GCE and GCE/MWCNTs display similar characteristics, without distinct current peaks. The considerably higher current values for the GCE/MWCNTs stem from the increased capacity at the electrode–solution interface. This augmentation results from the application of the MWCNT layer onto the relatively smooth GCE surface, leading to a significant surface development. The initial increase in the anodic current at a potential around +1.4 V correlates with the oxygen evolution reaction. However, the CV voltammograms for GCE/MWCNTs/PdNPs exhibit a markedly different profile compared with the GCE and GCE/MWCNTs (Figure 2B). This divergence in shape is directly linked to the presence of PdNPs. The increase in the anodic current observed from a potential of approximately +0.6 V corresponds to the oxidation of PdNPs. The electrooxidation process of Pd is intricate and can manifest through diverse pathways [14,15]. Principally, this process involves the passivation of Pd, leading to the formation of Pd(II) oxide (PdO). Concurrently, the cathodic peak detected at a potential ranging from +0.47 to +0.45 V relates to the reduction of PdO previously formed during the scanning in the anodic direction. Notably, an increase in the Pd quantity on the electrodes correlates with amplified peak currents, corresponding to the electrooxidation of Pd and the subsequent electroreduction of PdO.

**Figure 3:**
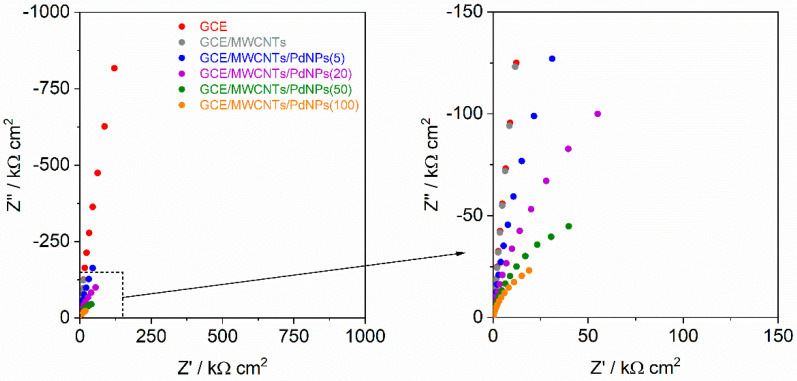
Impedance characteristics (Nyquist plots) recorded in 0.1 mol L^−1^ HClO_4_ solution for the GCE, GCE/MWCNTs, and GCE/MWCNTs/PdNPs. Frequency range of 10,000–0.01 Hz (amplitude 10 mV, 50 measuring points).

Subsequently, the EIS measurements were carried out within a frequency range of 10,000–0.01 Hz with a signal amplitude of 10 mV. The results of EIS measurements, depicted as Nyquist plots, are presented in Figure 3. The obtained EIS characteristics reveal a reduction in impedance following each modification of the GCE. The GCE/MWCNTs exhibited an impedance value of 1.7 × 10^5^ Ω cm^2^ at the lowest frequency (10 mHz), notably one order of magnitude lower than the impedance value for the unmodified GCE (1.3 × 10^6^ Ω cm^2^). However, for GCE/MWCNTs/PdNPs, the electrode resistance decreased with increasing Pd content. The impedance value for the GCE/MWCNTs/PdNPs(5) at 10 mHz was equal to 1.6 × 10^5^ Ω cm^2^, decreasing to 2.9 × 10^4^ Ω cm^2^ for the GCE/MWCNTs/PdNPs(100). This decrease in electrode impedance value indicates increased electrochemical activity, and the reduction–oxidation processes are anticipated to occur more readily on the electrode.

### 3.3. The Electrocatalytic Activity of Each Electrode in the Process of Formaldehyde Oxidation

**Figure 4:**
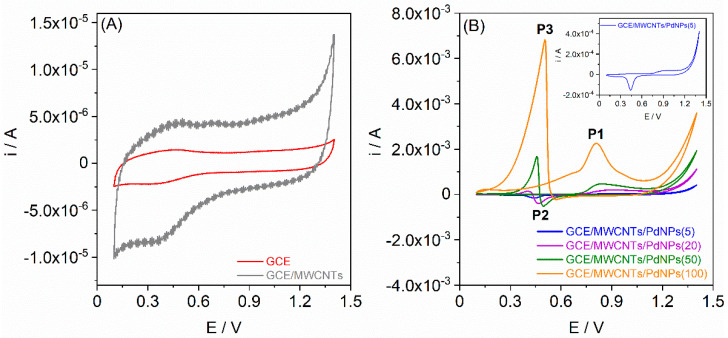
Cyclic voltammograms (scan rate of 100 mV s^−1^) recorded in 0.1 mol L^−1^ HClO_4_ solution with 0.1 mol L^−1^ formaldehyde for (**A**) GCE and GCE/MWCNTs, (**B**) GCE/MWCNTs/PdNPs.

The evaluation of the electrocatalytic activity of each of the GCE/MWCNTs/PdNPs with various amounts of deposited Pd was conducted using CV in a 0.1 mol L^−1^ HClO_4_ solution containing formaldehyde at a concentration of 0.1 mol L^−1^. The recorded cyclic voltammograms spanning the potential range from +0.1 V to +1.4 V with a potential scan rate of 100 mV s^−1^ are presented in Figure 4A for GCE and GCE/MWCNTs and in Figure 4B for GCE/MWCNTs/PdNPs.

As evident from Figure 4A, the shape of the cyclic voltammograms for the GCE and GCE/MWCNTs remain consistent with those in the HClO_4_ solution without formaldehyde (Figure 2A). In both cases, no current peaks are visible within the tested potential range, indicating the inactivity of these electrodes toward the electrooxidation of formaldehyde. In the presence of MWCNTs, the electrode surface becomes more developed resulting in an increase capacitive current. However, for the GCE/MWCNTs/PdNPs, there is a distinct difference in the shapes of the cyclic voltammograms (Figure 4B). Cyclic voltammograms for the GCE/MWCNTs/PdNPs(5) closely resemble those for the HClO_4_ solution (Figure 2B), suggesting that on an electrode with a small amount of deposited PdNPs, formaldehyde electrooxidation occurs to a limited extent. In contrast, the other GCEs modified with MWCNTs/PdNPs exhibit peak P1 in the cyclic voltammograms at a potential ranging from +0.81 to +0.92 V (Table 3), which is associated with the oxidation of both formaldehyde and PdNPs. Further polarization in the anodic direction leads to a decrease in current, attributed to the blocking of the PdNP surface by the adsorption of carbon monoxide (CO), an intermediate product of formaldehyde oxidation, on the electrode surface. Reversing the polarization to the cathodic direction results in the appearance of a cathodic peak P2 at a potential spanning from +0.57 to +0.47 V (Table 3), which is associated with the reduction of PdO. As a result of further cathodic polarization, a second anodic peak P3, commonly referred to as the “inversion” peak, emerges. This peak reaches its maximum at a potential occurring in the range from +0.50 to +0.40 V (Table 3). The appearance of this peak is attributed to the regained electroactivity of the PdNPs, leading to further formaldehyde oxidation due to CO desorption and possible conversion to carbon dioxide (CO_2_). This process unblocks the electrode surface previously hindered by adsorbed CO [59,60].

Based on the cyclic voltammograms, it can be concluded that the electrooxidation of formaldehyde involving PdNPs is a complex process that may follow various mechanisms [55,61]. The electrooxidation of formaldehyde can be represented by the reaction Equations (1)–(3):

(1) $$HCHO+H_{2}O⇄{CH}_{2}{\left(OH\right)}_{2}⟶{CO}_{2}+{4H}^{+}+{4e}^{-}$$

(2) $$HCHO⟶{CO}_{\left(ads\right)}+{2H}^{+}+{2e}^{-}$$

(3) $${CO}_{\left(ads\right)}+H_{2}O⟶{CO}_{2}+{2H}^{+}+{2e}^{-}$$

The enhanced mechanism of electrocatalytic performance of formaldehyde oxidation on the GCE modified with MWCNTs and PdNPs is attributed to several key factors. Pd, known for its catalytic activity in numerous organic reactions, plays a crucial role in this enhancement. The deposition of PdNPs with a nanopore cavity onto the electrode material significantly increases its electroactive surface area, providing more active sites for electrocatalysis [54,62]. The nanopores create a unique structure that facilitates a rapid and efficient electron transfer pathway from the exterior to the interior of the electrode. This is especially crucial for formaldehyde oxidation, where the accelerated electron transfer significantly enhances the overall electrocatalytic performance. Furthermore, the incorporation of MWCNTs contributes to the overall improvement. MWCNTs possess excellent electrical conductivity and mechanical strength, enhancing the electrical and structural properties of the modified electrode. This, in turn, promotes efficient charge transfer during electrocatalytic reactions [55,63]. In summary, the combination of PdNPs and MWCNTs synergistically amplifies the electrocatalytic performance of the modified GCE by increasing the electroactive surface area, improving electrical conductivity, and facilitating fast electron transfer through the nanopore cavity structure.

### 3.4. The Long-Term Stability Study

**Figure 5:**
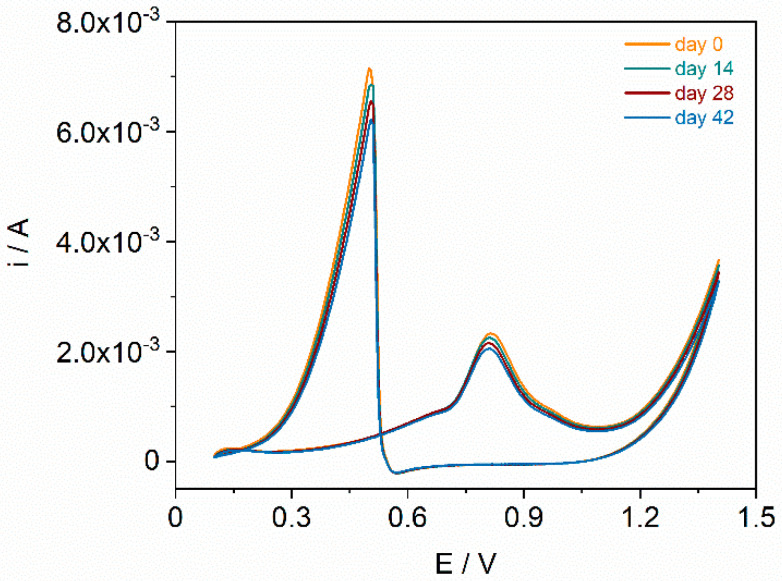
Cyclic voltammograms (scan rate of 100 mV s^−1^) recorded in 0.1 mol L^−1^ HClO_4_ solution with 0.1 mol L^−1^ formaldehyde for GCE/MWCNTs/PdNPs(100) over a period of 42 days.

The long-term stability of the developed electrode material showcasing the best electrocatalytic properties in the electrooxidation reaction of formaldehyde, i.e., GCE/MWCNTs/PdNPs(100), was investigated through cyclic voltammetric experiments in a 0.1 mol L^−1^ formaldehyde solution over a period of 42 days. Measurements were systematically conducted at 14-day intervals. As depicted in Figure 5, the cyclic voltammograms displayed minimal variations in peak currents, with a relative standard deviation value below 10%. These results indicate that the GCE/MWCNTs/PdNPs(100) exhibited satisfactory stability during the tested period.

## 4. Conclusions

This paper presents the complete procedure for preparing modified GCEs using MWCNTs and PdNPs, characterizing their morphology and electrochemical properties and demonstrating their electrocatalytic properties in the formaldehyde electrooxidation process. PdNPs were electrochemically obtained on a GCE covered with MWCNTs using a charge-controlled potentiostatic method, employing four different deposition charges (−5, −20, −50, and −100 mC). This allowed the preparation of four GCE/MWCNTs/PdNPs, each containing varying amounts of Pd (0.3, 1.1, 2.8, and 5.5 µg, respectively). The fabricated GCE/MWCNTs/PdNPs as well as unmodified GCE and GCE/MWCNTs underwent topographical characterization using AFM and SEM, as well as electrochemical assessment using CV and EIS. Based on the presented findings, the following conclusions were drawn:

(i) the amount of Pd deposited on the electrode depends on the charge utilized during electrodeposition: higher charges lead to a greater amount of Pd on the electrode;
(ii) the electrodeposition process produced PdNPs, and their size varied depending on the applied charge (amount of deposited Pd): a higher deposition charge (amount of deposited Pd) resulted in larger PdNPs;
(iii) small deposition charges result in surface smoothing, while very large deposition charges lead to a significant development of the surface;
(iv) the resistance of the GCE/MWCNTs/PdNPs was lower compared with that of the GCE and GCE/MWCNTs, and it decreased with an increase in the amount of Pd on the electrode;
(v) GCE and GCE/MWCNTs exhibited no electrocatalytic activity in the formaldehyde electrooxidation;
(vi) GCE/MWCNTs/PdNPs demonstrated electrocatalytic activity in the formaldehyde electrooxidation. This activity increased with the growth of PdNP size (amount of deposited Pd).

To conclude, the presented method of fabricating GCE/MWCNTs/PdNPs is promising for the development of electrochemical platforms suitable for the electrooxidation of various small organic molecules. The material produced may find applications as an electrochemical sensor, catalyst, or environmental remediation device for the removal of small organic molecules such as formaldehyde.

## Figures and Tables

**Table 1:** Determined topographic parameters characterizing the surface of the tested electrodes for scanning areas of 1 µm^2^.

Electrodes	Sr (μm^2^)	SAD (%)	Rq (nm)	Ra (nm)	Rmax (nm)
GCE	1.02	2.3	2.4	1.9	20.8
GCE/MWCNTs	1.89	88.9	25.9	20.3	204
GCE/MWCNTs/PdNPs(5)	1.51	50.7	29.9	23.1	241
GCE/MWCNTs/PdNPs(20)	1.48	48.2	26.7	20.8	218
GCE/MWCNTs/PdNPs(50)	1.89	88.5	51.0	40.8	304
GCE/MWCNTs/PdNPs(100)	1.81	81.2	49.2	39.2	308

**Table 2:** Quantitative elemental composition for each of the GCE/MWCNTs/PdNPs.

Electrodes	Mass Content (%)	
	C(K)	Pd(L)
GCE/MWCNTs/PdNPs(5)	90.15	9.85
GCE/MWCNTs/PdNPs(20)	72.99	27.01
GCE/MWCNTs/PdNPs(50)	28.29	71.71
GCE/MWCNTs/PdNPs(100)	11.32	88.48

**Table 3:** Peak potential and peak current values for the GCE/MWCNTs/PdNPs derived from CVs presented in Figure 4B.

Electrodes	P1		P2		P3	
	E (V)	i (A)	E (V)	i (A)	E (V)	i (A)
GCE/MWCNTs/PdNPs(5)	0.97	3.22 × 10^−5^	0.44	−1.43 × 10^−4^	-	-
GCE/MWCNTs/PdNPs(20)	0.92	1.92 × 10^−4^	0.47	−3.53 × 10^−4^	0.40	4.95 × 10^−4^
GCE/MWCNTs/PdNPs(50)	0.84	4.64 × 10^−4^	0.50	−4.82 × 10^−4^	0.46	2.16 × 10^−3^
GCE/MWCNTs/PdNPs(100)	0.81	2.02 × 10^−3^	0.57	−1.63 × 10^−4^	0.50	6.97 × 10^−3^

## Data Availability

The raw data supporting the conclusions of this article will be made available by the authors on request.
